# α-Glucosidase inhibitory activity of polyphenol-rich sugarcane extract: screening and mechanistic insights based on biolayer interferometry-mass spectrometry

**DOI:** 10.3389/fnut.2025.1575409

**Published:** 2025-07-29

**Authors:** Mengli Yao, Jia Liu, Fang Zhou, Haizhi Li, Ruoyong Wang, Zhong Han, Jie Liu, Wei Chen, Guoyu Liu, Shuheng Yang, Shenlin Duan, Xiaofeng Han, Peng Yuan

**Affiliations:** ^1^China National Research Institute of Food and Fermentation Industries Co., Ltd., Beijing, China; ^2^Air Force General Hospital PLA, Beijing, China; ^3^School of Food Science and Engineering, South China University of Technology, Guangzhou, China; ^4^Key Laboratory of Geriatric Nutrition and Health, Beijing Technology and Business University, Ministry of Education, Beijing, China; ^5^Peking Union Medical College Hospital, Beijing, China

**Keywords:** polyphenol-rich sugarcane extract, α-glucosidase inhibition, bioactive compounds, BLI-MS, molecular docking

## Abstract

**Introduction:**

Polyphenol-rich sugarcane extract (PRSE) contains bioactive compounds with potential hypoglycemic properties, but its direct interaction with α-glucosidase has not been explored.

**Methods:**

This study investigated the inhibitory mechanism of PRSE on α-glucosidase using enzyme kinetics. Bioactive compounds with α-glucosidase-binding affinity were identified through biolayer interferometry-mass spectrometry (BLI-MS), and the binding mechanisms were further explored via molecular docking analysis.

**Results and discussion:**

PRSE was found to inhibit α-glucosidase through a mixed-type mechanism. A total of 29 compounds, including 4 coumarins, 9 phenolic acids, and 16 flavonoids, were identified in the PRSE dissociation solution. Representative compounds included coumarin, kaempferol, apigenin 7-o-neohesperidoside, and vicenin 3. Notably, apigenin 7-o-neohesperidoside and vicenin 3 were identified for the first time as potential α-glucosidase inhibitors.These compounds interacted with key residues of α-glucosidase, such as Asp and Glu, via hydrogen bonding, π-anion interactions, and hydrophobic forces. These findings suggest that PRSE could serve as a promising natural source of α-glucosidase inhibitors. The application of BLI-MS proved effective for screening target bioactive compounds in plant extracts. PRSE may have potential applications in functional foods for postprandial glycemic control and type 2 diabetes prevention.

## 1 Introduction

Controlling the postprandial rise in blood glucose levels is essential for the prevention of diabetes (DM) and its complications. The two glycoside hydrolases, α-amylase and α-glucosidase, play pivotal roles in regulating the digestion and absorption of carbohydrates (1, 2). Specifically, α-glucosidase hydrolyzes the glycosidic bonds at the non-reducing ends of oligosaccharides to release glucose. Inhibiting its activity can effectively delay glucose release, making it a promising strategy for controlling postprandial blood glucose (3). Acarbose, miglitol, and voglibose are commonly used α-glucosidase inhibitors in the clinical treatment of DM; however, due to their side effects, there is an urgent need to identify new plant-derived therapeutic agents for DM management (4, 5).

Plant extracts have long been recognized as valuable resources in drug development. Since 1981, approximately two-thirds of new small-molecule drugs have been derived from plant extracts, their derivatives, or mimics (6, 7). Exploring active components in plant extracts and elucidating their bioactivities has become a hot topic in pharmaceutical research (7, 8). Among these, polyphenolic compounds found in plant extracts have gained significant attention in drug development due to their rich bioactivities (9). Studies have shown that polyphenolic compounds, such as apigenin, rutin, kaempferol, and curcumin, exhibit significant inhibitory effects on α-glucosidase. Compared to clinical drugs like acarbose, natural inhibitors have the advantage of fewer side effects (7).

Recent studies have highlighted sugarcane and its byproducts, such as molasses and bagasse, as promising natural sources rich in polyphenolic compounds with significant bioactivities (10–12). Several investigations have explored the hypoglycemic effects of polyphenol-rich sugarcane extract (PRSE) and its underlying mechanisms. For instance, Zheng et al. (10) identified phenolic compounds such as tricin 4-O-guaiacylglyceryl ether-7-O-glucopyranoside, genistin, p-coumaric acid, and quercetin from 30% sugarcane bagasse extract using UHPLC-HR-TOFMS. These compounds were confirmed to exhibit hypoglycemic and enzyme inhibitory effects. Similarly, Zhao et al. (13) analyzed sugarcane bagasse extracts via HPLC and identified major phenolic acids, including gallic acid, ferulic acid, coumaric acid, and chlorogenic acid, which also displayed hypoglycemic activity. Additionally, Deseo et al. (14) utilized LC-MS to detect flavonoids in sugarcane molasses, including apigenin-C-glycosides, methoxyluteolin-C-glycosides, and tricin-O-glycosides, which were considered potential α-glucosidase inhibitors. Collectively, these studies demonstrate that polyphenolic compounds in sugarcane and its byproducts hold considerable promise as natural agents for controlling blood glucose levels, providing new avenues for diabetes treatment.

While the hypoglycemic effects of PRSE are well-documented, the precise mechanisms by which it inhibits α-glucosidase remain unclear.. To investigate this, the present study combines biolayer interferometry (BLI) and mass spectrometry (MS). BLI, a label-free detection technique based on optical interference,.enables real-time monitoring of molecular interactions. However, identifying specific molecules within complex mixtures poses challenge (6). By integrating BLI with MS (BLI-MS), this approach enhances detection sensitivity and specificity, making it particularly advantageous for high-throughput screening of bioactive compounds in natural products (15, 16).

This study systematically investigated the inhibitory kinetics of PRSE against α-glucosidase. Active inhibitors were identified using BLI-MS, and molecular docking revealed their mechanisms of action. These methods provide an efficient framework for screening bioactive compounds in plant extracts. The identification of natural α-glucosidase inhibitors in PRSE highlights its potential as a promising, low-side-effect alternative for managing type 2 diabetes, with applications in functional foods and dietary supplements.

## 2 Materials and methods

### 2.1 Materials and chemicals

Polyphenol-rich sugarcane extract (PRSE) was procured from Qingyunshan Pharmaceutical Co., Ltd., China. α-Glucosidase (32.4 U/mg, from *Saccharomyces cerevisiae*) was sourced from Shanghai Yuanye Biotechnology Co., Ltd., China. G-MM-IGT biotin (Genemor) was obtained from Jiangsu Lesai Biotechnology Co., Ltd., China. SSA biosensors for biolayer interferometry (BLI) analysis were procured from Sartorius, Germany. P-Nitrophenyl α-D-glucopyranoside (PNPG), acarbose, gallic acid, and rutin were acquired from Sigma-Aldrich Co., Ltd., United States, and Tween 20 from Beyotime Biotechnology Co., Ltd., China. All other chemicals were of analytical grade, and freshly prepared ultrapure water was used in all experiments.

### 2.2 Determination total flavonoid content (TFC)

The total flavonoid content was determined based on the method described by Zheng et al. (17), with minor modifications to sample concentration and reaction volumes to better suit the PRSE matrix. Briefly, a standard curve was prepared by dissolving 24.1 mg of rutin in 60% ethanol and diluting it to 50 mL (482 μg/mL); the solution was stored at 4°C. Aliquots of 0.2, 0.4, 0.6, 0.8, and 1.0 mL were taken, followed by the addition of 150 μL sodium nitrite and incubation for 6 min. Subsequently, 150 μL of 10% Al(NO*3*)*3* was added, and the mixture was incubated for another 6 min. Finally, 2 mL of 4% NaOH was added, and the mixture was diluted to 5 mL with 60% ethanol, mixed, and incubated for 15 min before measuring the absorbance at 510 nm. A 1 mL aliquot of the PRSE solution (2 mg/mL) was treated using the same procedure, and the absorbance was used to calculate the flavonoid content based on the rutin standard curve.

### 2.3 Determination total phenolic content (TPC)

The total phenolic content was determined based on the method described by Zheng et al. (17), with minor modifications to reagent concentrations, reaction times, and sample preparation to accommodate the PRSE matrix. A 1 mg/mL gallic acid stock solution was prepared, and aliquots of 0.1–0.6 mL were diluted to 10 mL with ultrapure water to prepare working solutions of 10–60 μg/mL. For each working solution and the PRSE sample (2 mg/mL), 1 mL was mixed with 5 mL of 10% Folin–Ciocalteu reagent, vortexed, and allowed to stand for 6 min to ensure full color development. Subsequently, 4 mL of 7.5% Na_2_CO_3_ solution was added, and the mixture was left to stand for 40 min. The addition of sodium carbonate created an alkaline environment (pH > 10), which is optimal for the redox reaction between phenolic compounds and the Folin–Ciocalteu reagent. Absorbance was measured at 765 nm, and the total phenolic content was calculated using the gallic acid standard curve.

### 2.4 α-Glucosidase inhibition assay

The method described by Ren et al. (18) was modified slightly for this study, with adjustments to the PRSE solution concentrations and incubation time to better suit the experimental setup. Specifically, a 200 μL aliquot of PRSE solution at different concentrations (10, 20, 30, 40, 60, and 120 μg/mL) was mixed with 200 μL of α-glucosidase working solution, and the mixture was incubated at 37°C for 5 min. Subsequently, 200 μL of PNPG (2.5 mmol/L) was added and mixed thoroughly, followed by incubation at 37°C for an additional 15 min. The reaction was then terminated by adding 800 μL of Na_2_CO_3_ (0.2 mol/L). A 200 μL aliquot of the reaction mixture was transferred to a 96-well plate, and the absorbance at 405 nm was measured using a microplate reader. Acarbose, at concentrations of 0.01, 0.015, 0.02, 0.025, and 0.03 μg/mL, was used as a positive control. Each sample was tested in triplicate. The α-glucosidase inhibition rate was determined using Equation 1:


(1) $$Inhibitoryrates\left(\%\right)=\frac{O\,D_{t\,e\,s\,t}-O\,D_{b\,l\,a\,n\,k}}{c\,o\,n\,t\,r\,o\,l\,O\,D_{t\,e\,s\,t}-c\,o\,n\,t\,r\,o\,l\,O\,D_{b\,l\,a\,n\,k}}$$

### 2.5 α-Glucosidase inhibition kinetics

Following the method of Sun et al. (19) with slight modifications, the substrate concentration (PNPG) was fixed at 2.5 mmol/L, while α-glucosidase concentrations were adjusted to 0.2, 0.4, 0.6, 0.8, and 1.0 U/mL. Initial reaction rates (ΔOD/min) were measured across varying PRSE concentrations (0, 60, 120, 200 μg/mL), and a rate *vs*. enzyme concentration curve was constructed to evaluate the reversibility of PRSE’s inhibition of α-glucosidase.

To determine the inhibition type and constants, Lineweaver-Burk plots were employed. With α-glucosidase fixed at 1.5 U/mL, PNPG concentrations were varied (0.5, 1.0, 2.0, 2.5, and 5.0 mmol/L), and reaction rates (ΔOD/min) were recorded at PRSE concentrations of 0, 10, 20, 50, and 60 μg/mL. The α-glucosidase concentration (1.5 U/mL) was selected based on preliminary experiments to ensure sufficient enzymatic activity within the linear range of detection, while PRSE concentrations were chosen to represent a range from low to high inhibition levels observed in prior dose–response assays. The reciprocal of initial reaction rates (1/V) was plotted against the substrate concentrations (1/S), and the Michaelis constant (K_*m*_) and maximum velocity (V_*max*_) were derived to classify the inhibition type (Equation 2).


(2) $$\frac{1}{V}=\frac{1}{V_{m\,a\,x}}+\frac{K_{m}}{V_{m\,a\,x}}\times \frac{1}{\left[I\right]}$$

Where, V is the initial reaction velocity; [I] is the concentration of PRSE.

The inhibition constants of PRSE for the free enzyme (K_*I*_) and for the enzyme-substrate complex (K_*IS*_) were derived from Equations 3 and Equations 4.


(3) $$S\,l\,o\,p\,e=\frac{K_{m}}{V_{m\,a\,x}}+\frac{K_{m}\,\left[I\right]}{V_{m\,a\,x}\,K_{I}}$$

(4) $$Y-i\,n\,t\,e\,r\,c\,e\,p\,t=\frac{1}{V_{m\,a\,x}}\,\left(1+\frac{\left[I\right]}{K_{I\,S}}\right)$$

### 2.6 Biolayer interferometry

**FIGURE 1:**
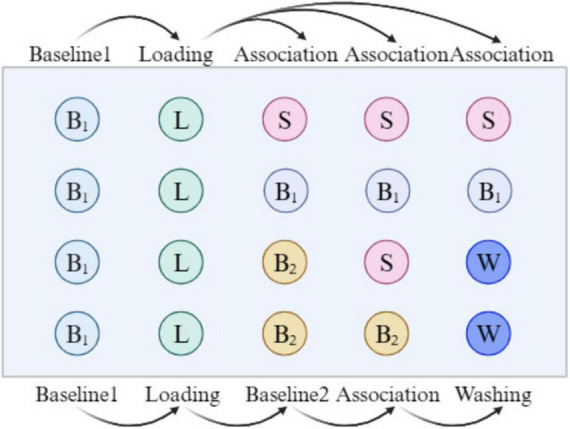
A brief flow chart of the BLI experiment.

Based on the methods of Guo et al. (15) and Zhou et al. (20), with slight modifications, BLI kinetic analysis and fishing experiments were performed for PRSE, as illustrated in Figure 1. α-Glucosidase (2 mg/mL) was dissolved in PBS and biotinylated using the G-MM-IGT reagent. The biotinylated enzyme solution with the highest concentration was collected for further testing. In a 96-well plate, 200 μL PBS was added to the B_1_ wells for baseline1 signal recording, and 200 μL of the biotinylated α-Glu solution was added to the L wells. Baseline1 (60 s) and Loading (300 s) were performed to immobilize α-Glu onto the SSA sensor, and the immobilization signal was recorded. For kinetic analysis, 200 μL of PRSE solutions at different concentrations (200, 300, 400, 500, 600 μg/mL) were added to the S wells in the first row, while PBS was added to the B_1_ wells in the second row as a blank control. The process included baseline1 (30 s) and association (300 s), and the association signals were recorded.

In the fishing experiment for active compounds, 200 μL PBS (containing 0.1% Tween 20) was added to the B_2_ wells in the 3rd and 4th rows, while 200 μL of PRSE solution (2 mg/mL in 0.1% Tween 20 PBS) was added to the S wells in the 3rd row, and 200 μL of 0.1% formic acid was added to the W wells in the 3rd and 4th rows for washing. A total of 30 cycles were conducted, including baseline2 (30 s), association (300 s), and washing (20 s), to complete the fishing of active compounds. Data were collected and analyzed using the ForteBio Octet system (version 11.x). The eluates without PRSE were defined as S_1_, the eluates with PRSE as S_2_, and thePRS solutions not subjected to BLI as S_3_. Each group was tested in triplicate.

### 2.7 MS analysis

The S_1_, S_2_, and S_3_ sample groups were analyzed using an ultra-high-performance liquid chromatography (UHPLC) system. Analysis was performed on a Vanquish UHPLC system (Thermo Fisher Scientific) with a Phenomenex Kinetex C_18_ column (2.1 mm × 50 mm, 2.6 μm) for chromatographic separation of target compounds. The mobile phases consisted of an aqueous phase (Phase A, containing 0.01% acetic acid) and an organic phase (Phase B, isopropanol : water = 1:1, v/v). The sample tray was maintained at 4°C, and the injection volume was set to 2 μL. After chromatographic separation, primary and secondary mass spectrometric data were collected using an Orbitrap Exploris 120 mass spectrometer controlled by Xcalibur software (version 4.4, Thermo Fisher Scientific). MS parameters were set as follows: Sheath gas flow rate, 50 Arb; auxiliary gas flow rate, 15 Arb; capillary temperature, 320°C; full-scan MS resolution, 60,000; MS/MS resolution, 15,000; collision energy set at stepped normalized collision energy levels of 20/30/40; and spray voltage of 3.8 kV in positive mode and –3.4 kV in negative mode.

### 2.8 Calculation of the relative binding amount

The relative binding amount (RBA) was calculated after analyzing the samples using BLI and UHPLC. The analysis involved three solutions: The dissociation buffer of the PRSE-free solution (PBS + 0.1% Tween 20) after BLI analysis (S_1_), the dissociation buffer of the PRSE solution after BLI analysis (S_2_), and the PRSE solution without BLI analysis (S_3_). Compounds detected in both S_2_ and S_3_ but absent in S_1_, based on mass spectrometry analysis, were identified as potential PRSE compounds with binding affinity to α-glucosidase. The calculation formula is provided in Equation 5.


(5) $$RBA\left(\%\right)\frac{\mathrm{Peak}\,\mathrm{area}\,\mathrm{of}\,\mathrm{the}\,\mathrm{compounds}\,\mathrm{in}\,S_{2}}{\mathrm{Peak}\,\mathrm{area}\,\mathrm{of}\,\mathrm{the}\,\mathrm{compounds}\,\mathrm{in}\,S_{3}}\times 100$$

### 2.9 Molecular docking

Molecular docking simulations were conducted following the modified method of Lin, involving the potential compounds (structures shown in Supplementary Figure S1) and α-glucosidase (PDB ID 3AJ7, derived from *S. cerevisiae*) (21). The selected compounds represent key bioactive constituents identified or predicted from PRSE, based on previous reports and preliminary fishing results. The 2D structures of these PRSE-derived compounds were generated using ChemDraw, converted to 3D in Chem3D, and subjected to hydrogen atom addition, charge assignment, and energy minimization before being saved as mol_2_ files. The α-glucosidase crystal structure was retrieved from the PDB database. Water molecules were removed, and hydrogen atoms and charges were added using AutoDock, after which the structure was saved as pdbqt files. Docking simulations were carried out using default parameters to determine the lowest binding free energy. Discovery Studio 4 was utilized for the visualization of protein-ligand interactions.

### 2.10 Statistical analysis

All experimental data were measured in triplicate and reported as the mean ± standard deviation (*n* = 3). Graphs were generated using Origin Pro 9.0 and GraphPad Prism 9.0 software.

## 3 Results and discussion

### 3.1 TPC and TFC of PRSE

Polyphenolic compounds are well-documented for their diverse biological activities, particularly in glycemic regulation. Polyphenols are categorized into flavonoids, phenolic acids, stilbenes, and lignins based on the number of phenolic rings and structural variations (22). Studies have demonstrated that polyphenols mitigate postprandial blood glucose elevation through multiple mechanisms, such as inhibiting α-amylase and α-glucosidase activities, suppressing intestinal glucose absorption, stimulating insulin secretion, and reducing hepatic glucose output (23). Li et al. (24) demonstrated that mung bean polyphenols, including quinic acid, apigenin, and vitexin, exhibit significant *in vitro* inhibitory effects on α-glucosidase and effectively lower blood glucose levels while improving insulin resistance in type 2 diabetic mice. Similarly, Chen et al. (25) reported that active plant compounds, including flavonoids, alkaloids, polysaccharides, and polyphenols, form the molecular basis for the hypoglycemic effects of mulberries.

PRSE was found to be rich in phenolic acids and flavonoids, with TFC of 53.8 ± 1.6 mg CE/g and TPC of 218.3 ± 2.4 mg GAE/g (Table 1), exceeding or comparable to values reported for other sugarcane extracts. Deseo et al. (14) reported a TPC of 205 mg GAE/g and a TFC of 55 mg CE/g in ethanol extracts of sugarcane molasses, while Ji et al. (11) observed a TPC exceeding 200 mg GAE/g in hydrophobic sugarcane molasses extracts. These abundant polyphenvolic compounds likely contribute to PRSE’s inhibitory activity against α-glucosidase, aligning with hypoglycemic mechanisms observed in other plant extracts and reinforcing PRSE’s potential as a hypoglycemic agent (26). In addition to postprandial glucose regulation, such compounds may also support broader aspects of glucose metabolism, including improved insulin sensitivity and reduced glucose absorption.

**TABLE 1:** TPC and TFC of PRSE and its inhibitory activity on *α-*Glucosidase.

Sample	TFC (mg CE/g)	TPC (mg GAE/g)	IC_50_ (μ g/mL)
PRSE	53.8 ± 1.6	218.3 ± 2.4	79 ± 6.1
Acarbose	N/A	N/A	0.018 ± 0.02

Acarbose is included for comparison, but no data for TFC and TPC were measured; hence, the corresponding cells are marked as “N/A” (Not Applicable).

### 3.2 Inhibition of α-glucosidase

This study assessed the inhibitory activity of PRSE against α-glucosidase, with acarbose serving as the positive control. Acarbose exhibited strong α-glucosidase inhibition, with an IC_50_ of 0.021 ± 0.02 μg/mL (Table 1). In comparison, PRSE showed notable inhibition, with an IC_50_ of 79 ± 6.1 μg/mL (27).

**FIGURE 2:**
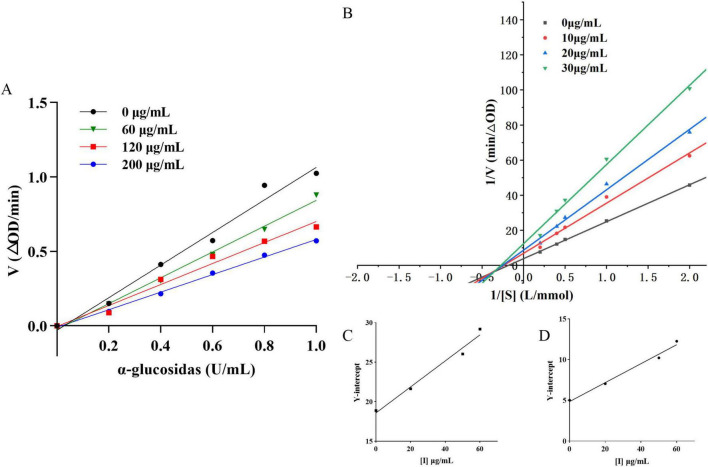
The inhibitory effect of PRSE on *α-*glucosidase. **(A)** Kinetic curve of the inhibitory effect of PRSE on *α-*glucosidase; **(B)** Lineweaver-Burk plot of the inhibitory effect of PRSE on *α-*glucosidase. The inset shows the relationship between the slope **(C)** and the Y-intercept **(D)** with PRSE concentration.

Initial reaction velocities plotted against enzyme concentrations (Figure 2A) showed lines intersecting at the origin, with slopes decreasing as PRSE concentrations increased. These findings indicate that PRSE exerts reversible inhibition by reducing enzyme activity without affecting the amount of active enzyme, which is consistent with the inhibition patterns observed for other plant extracts (21). In reversible inhibition, increasing the enzyme concentration can restore reaction velocity proportionally, which is consistent with the observed linearity through the origin—indicating that the inhibitor does not permanently inactivate the enzyme (28). The Lineweaver-Burk double-reciprocal plot (Figure 2B; Table 2) revealed that increasing PRSE concentrations reduced V_*max*_ and K_*m*_, indicating enhanced enzyme-substrate affinity. Furthermore, the intersection of all lines in the third quadrant indicates a mixed-type inhibition mechanism. This inhibition mechanism is consistent with that observed for polyphenols from mung bean hull dietary fiber and passion fruit peel, which also target α-glucosidase (18, 19, 29).

**TABLE 2:** Inhibition kinetics parameters of *α-*glucosidase by PRSE.

Sample concentration (μ g/mL)	Equation	R^2^	K_*m*_ (μ g/mL)	V_*m*_ (Abs/min)
0	*y* = 18.87x+5.02	0.99368	3.76	0.20
10	*y* = 21.63x+7.04	0.98494	3.07	0.14
50	*y* = 26.03x+10.22	0.94804	2.55	0.10
60	*y* = 29.16x+12.26	0.99637	2.38	0.08

Secondary fitting curves derived from Lineweaver-Burk slope and intercept data (Figures 2C,D) produced binding constants of PRSE for the free enzyme (K_*I*_) and enzyme-substrate complex (K_*IS*_) as 0.018 μg/mL and 0.20 μg/mL, respectively. These findings confirm PRSE’s strong α-glucosidase inhibitory activity and show its higher binding affinity to the enzyme-substrate complex than to the free enzyme (30). R^2^ values of 0.9862 and 0.9796, derived from linear regression analysis, suggest a relatively simple binding mode between PRSE and the enzyme, likely involving a primary binding site (17). In conclusion, PRSE demonstrates potent α-glucosidase inhibition activity, highlighting its potential as a food-based source of α-glucosidase inhibitors (31).

### 3.3 Screening of α-glucosidase binding components in PRSE

**FIGURE 3:**
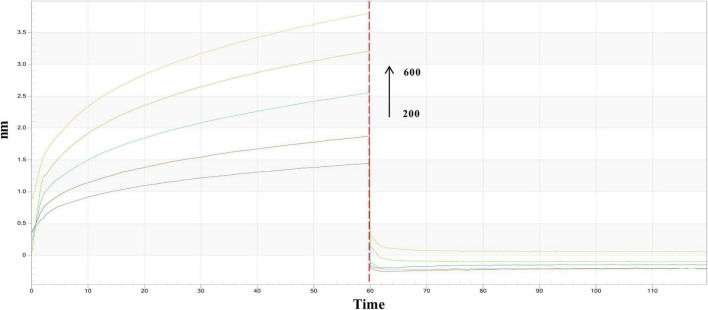
Real-time kinetic binding sensorgrams of different concentrations of PRSE increasing from 200 to 600 μg/mL are shown. Response (nm) indicates the optical thickness on the SSA biosensor layer.

This study further validated the interaction between PRSE and α-glucosidase by analyzing binding kinetics through real-time binding experiments. Association-dissociation curves showed a concentration-dependent increase in affinity between PRSE and α-glucosidase, confirming a direct and reversible interaction (Figure 3). Kinetic parameters — dissociation constant (K_*D*_), association rate constant (K_*on*_), and dissociation rate constant (K_*dis*_) — were calculated using ForteBio analysis software (version 11.x). The K_*D*_ value quantifies the binding affinity between a sample and its target; lower K_*D*_ values signify higher affinity, requiring less analyte to achieve 50% of maximum binding. K_*on*_ represents the binding rate; higher K_*on*_ values indicate faster and stronger binding. K_*dis*_ represents the dissociation rate; lower K_*dis*_ values suggest greater binding stability and reduced dissociation likelihood. Results revealed that PRSE and α-glucosidase exhibited a K_D_ of 6.19 × 10^−5^μM, K_on_ of 2.12 × 10^5^ 1/M⋅s, and K_dis_ of 1.31 1/s (Table 3). These findings suggest that PRSE exhibits strong affinity, rapid binding, and stable interactions with α-glucosidase (15).

**TABLE 3:** The main detected components in PRSE and their RBA (%) with *α-*glucosidase.

Compd.	MS_2_ name	mz	rt	Formula	Class	RBA%
1	Coumarin	147.0437	162.5	C_9_H_6_O_2_	Coumarins and their derivatives	13.28336883
2	Kaempferol	287.0542	210.9	C_15_H_10_O_6_	Flavonoids	10.74688552
3	Apigenin 7-O-neohesperidoside	601.151	181.3	C_27_H_30_O_14_	Flavonoids	10.33620329
4	Vicenin 3	565.1539	174	C_26_H_28_O_14_	Flavonoids	8.346978657
5	Ferulate	177.0541	162.6	C_10_H_10_O_4_	Phenolic acids	5.897128534
6	4-Hydroxycoum.Darin	163.0385	205.5	C_9_H_6_O_3_	Coumarins and their derivatives	5.89543992
7	Ombuoside	639.1905	181.8	C_29_H_34_O_16_	Flavonoids	4.906688327
8	Meloside A	595.1644	171.4	C_27_H_30_O_15_	Flavonoids	4.345383841
9	Iristectorin B	493.133	185.3	C_23_H_24_O_12_	Isoflavones	3.200242676
10	Scopoletin	193.049	172.2	C_10_H_8_O_4_	Coumarins and their derivatives	3.021664391
11	Luteolin	285.0404	208.3	C_15_H_10_O_6_	Flavonoids	2.630693284
12	Homoplantaginin	461.1089	156	C_22_H_22_O_11_	Flavonoids	2.606857373
13	Scoparone	207.0647	193.1	C_11_H_10_O_4_	Coumarins and their derivatives	2.519499166
14	Galangin	271.0595	186.1	C_15_H_10_O_5_	Flavonoids	2.492365513
15	Caffeic acid	179.0349	147.9	C_9_H_8_O_4_	Phenolic acids	1.934428269
16	Isopropyl ferulate	219.101	195	C_13_H_16_O_4_	Phenolic acids	1.813754175
17	Apigenin	269.0454	219	C_15_H_10_O_5_	Flavonoids	1.726469521
18	4-Methoxycinnamic acid	177.0556	211.4	C_10_H_10_O_3_	Phenolic acids	1.723205359
19	3,5-Dimethoxycinnamic acid	191.0697	220.8	C_11_H_12_O_4_	Phenolic acids	1.620526298
20	2-Methoxycinnamic acid	177.0556	174.1	C_10_H_10_O_3_	Phenolic acids	1.508415493
21	Glycitin	447.1277	179.8	C_22_H_22_O_10_	Isoflavones	1.258475053
22	Lonicerin	593.1517	169.5	C_27_H_30_O_15_	Flavonoids	1.074292611
23	Linarin	593.1848	173.9	C_28_H_32_O_14_	Flavonoids	0.907970314
24	Trans-Cinnamate	164.0717	206.7	C_9_H_8_O_2_	Phenolic acids	0.844409835
25	3,4,5-Trimethoxycinnamic acid	237.0767	194.5	C_12_H_14_O_5_	Phenolic acids	0.723494901
26	Ononin	431.1328	189.8	C_22_H_22_O_9_	Isoflavones	0.685890645
27	4-Hydroxycinnamic acid	163.04	180.6	C_9_H_8_O_3_	Phenolic acids	0.371175519
28	Liquiritin	417.1189	193	C_21_H_22_O_9_	Flavonoids	0.24800124
29	Naringin	601.1626	132.9	C_27_H_32_O_14_	Flavonoids	0.1172887

### 3.4 MS analysis

This study hypothesized that active small molecules in PRSE bind to biotinylated α-glucosidase and are collected in the dissociation solution, whereas molecules lacking affinity remain in the original solution. The binding components were identified via mass spectrometry, using unprocessed PRSE solution (not analyzed by BLI) as a reference. Relative binding amounts (RBA) were calculated to screen potential *α-*glucosidase inhibitors, with higher RBA values indicating greater efficacy (15).

Table 3 lists the retention times, accurate masses, molecular weights, chemical nameds, molecular formulas, and RBA values of the detected components. A total of 29 compounds were identified in the PRSE dissociation solution, including 4 coumarins and their derivatives, 9 phenolic acids and their derivatives, and 16 flavonoids. Ten compounds with strong binding affinities (RBA > 3%) were selected, including Coumarin, Kaempferol, Apigenin 7-O-neohesperidoside, Vicenin 3, Ferulate, 4-Hydroxycoumarin, Ombuoside, Meloside A, and Iristectorin B (6).

Certain active compounds from PRSE have known *α-*glucosidase inhibitory activities. For instance, Coumarin and its derivatives are well-established *α-*glucosidase inhibitors (32, 33). Zhang et al. (32) reported several synthesized coumarin derivatives exhibiting significant *α-*glucosidase inhibition, with IC_50_ values below 0.1 μg/mL. Furthermore, flavonoids like Kaempferol, Ferulate, and Vitexin, and their derivatives, have been identified in Hibiscus pollen and Sophora-derived polyphenols, confirming their potential as *α-*glucosidase inhibitors (26, 27, 34). However, compounds like Apigenin 7-O-neohesperidoside, Vicenin 3, and Ombuoside have not been previously reported for *α-*glucosidase inhibitory activities. This study uniquely identified these compounds as potential inhibitors through the BLI-MS integrated approach, highlighting their novelty. These findings align with prior studies and expand PRSE’s chemical profile as a source of antidiabetic functional components.

### 3.5 Molecular docking

**FIGURE 4:**
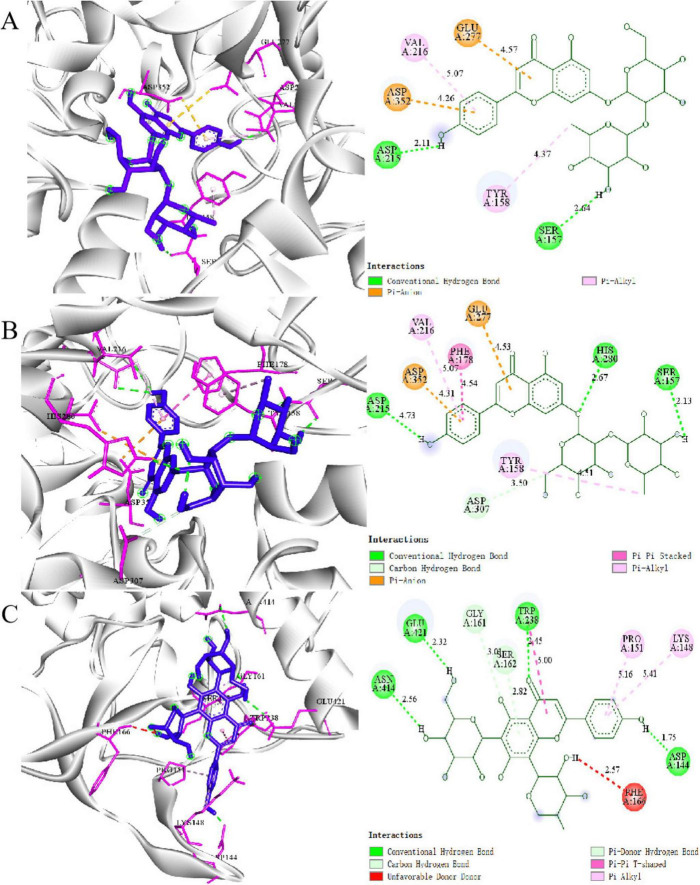
Molecular docking results: 2D and 3D structures of **(A)** Coumarin, **(B)** Apigenin 7-O-neohesperidoside, and **(C)** Vicenin 3 with *α-*glucosidas.

Molecular docking analysis was conducted to characterize the binding sites, binding energies, and interaction forces of the active compounds screened from PRSE with *α-*glucosidase (30, 35). Figure 4 shows the 3D and 2D docking results of the 10 active compounds with *α-*glucosidase. The docking results reveal that these small-molecule active compounds stabilize enzyme-ligand complexes by interacting with various amino acid residues (e.g., Asp, Glu, Tyr) within the *α-*glucosidase active pocket through van der Waals forces, hydrogen bonds, and carbon-hydrogen bonds (6).

Coumarin widely reported for its *α-*glucosidase inhibitory effects in previous studies, was analyzed as a reference compound (Figure 4A). Coumarin primarily interacts with Asp215 and Sep157 through hydrogen bonds and stabilizes its binding to Glu277 and Asp352 via π-anion interactions. Its aromatic ring structure further enhances hydrophobic interactions with Val216 and Tyr158. These critical residues (e.g., Asp, Glu) align with those commonly reported in the literature as essential for enzyme-inhibitor interactions (18, 36).

Compounds such as Apigenin 7-O-neohesperidoside and Vicenin 3, which exhibited high RBA values in the mass spectrometry screening, have not been previously reported to interact with *α-*glucosidase. Apigenin 7-O-neohesperidoside interacts with Asp307, Arg315, and Glu277 at the enzyme’s active site through multiple hydrogen bonds. The glycosyl moiety further stabilizes the binding through hydrophobic interactions with surface residues of the enzyme (Figure 4B). Similarly, Vicenin 3 forms hydrogen bonds between its glycosyl moiety and Lys155 and Asp352, while its aglycone structure interacts with Glu277 and Asp215 in the active pocket via π-anion interactions (Figure 4C). And the molecular docking results of other compounds with *α-*glucosidase are shown in Supplementary Figure S2.

The binding affinities of the 10 active compounds with *α-*glucosidase ranged from –11.0 to –6.8 kcal/mol (Table 4). Among these, Coumarin demonstrated the strongest affinity for *α-*glucosidase, with the lowest binding energy. Apigenin 7-O-neohesperidoside and Vicenin 3 exhibited significant affinities, with binding energies of –10.9 and –8.3 kcal/mol, respectively. For comparison, the binding affinity of the standard *α-*glucosidase inhibitor acarbose with *α-*glucosidase is reported to be –8.1 kcal/mol (Supplementary Table S1). These findings suggest that the active compounds identified in PRSE exhibit similar or even stronger affinities than the standard inhibitor, highlighting their potential as *α-*glucosidase inhibitors. The results align with BLI-MS screening, further validating the inhibitory activity of PRSE’s active compounds against *α-*glucosidase.

**TABLE 4:** Molecular docking parameters with active ingredients.

ID	Ligands	Affinity (kcal/mol)	Number of HBs	Number of closest residues	Interacting residues
1	Coumari	–11.0	3	6	Asp352,Val216, Glu277, Asp215, Tyr158 and Ser157
2	Kaempferol	–8.3	1	2	Tyr158 and Arg315
3	Apigenin 7-O-neohesperidoside	–10.9	3	7	Asp215, Tyr158, Ser157, Glu277, Phe178, Asp352 and Asp307
4	Vicenin 3	–8.3	5	10	Glu421, Asn414, Gly161, Trp238, Ser162, Phe166, Lys148, Asp144, Pro151 and Asp352
5	Ferulate	–8.7	2	10	Glu271, Lys13, Ala292, Trp15, Arg270, Glu296, Ile272, Asn259, His295 and Ile296
6	4-Hydroxycoum	–6.8	2	5	Asn235, Lys156, Phe420, Ile419 and Ala418
7	Ombuoside	–10.7	2	10	Asp215, Glu411, Phe303, Phe314, Arg315, Asp307, His280, Pro312, Tyr158 and Val216
8	Meloside A	–8.9	2	9	Glu271, Lys13, Trp15, Ala292, Glu296, Asn259, Arg270, Ile272 and Phe314
9	Iristectorin B	–8.8	2	7	Thr310, Pro312, His280, Leu313, Gly161, Asp307 and Arg315
10	Scopoletin	–7.5	2	8	Gly161, Lys156, Phe314, Ile419, Glu429, His423, Ala418 and Asn259

## Conclusion

This study systematically analyzed the enzyme inhibition kinetics of PRSE and demonstrated its significant mixed-type inhibitory effects on α-glucosidase. These findings highlight PRSE as a promising natural source of α-glucosidase inhibitors with potential applications in postprandial glycemic control. Using a BLI-MS integrated screening strategy, 29 active compounds were identified, predominantly phenolic acids and flavonoids. Among these, compounds such as coumarin, kaempferol, and vicenin 3 showed strong binding affinities, while apigenin 7-O-neohesperidoside was identified for the first time as a potential inhibitor. Molecular docking further confirmed interactions between these compounds and key residues (e.g., Asp and Glu) in the enzyme’s active pocket, stabilized by hydrogen bonding and hydrophobic forces.

These findings validate the reliability of the BLI-MS screening approach and demonstrate its effectiveness in identifying target compounds from complex plant extracts. Looking forward, *in vitro* and *in vivo* studies are warranted to confirm the hypoglycemic effects of these compounds and elucidate their mechanisms of action. Further work should also explore structure-activity relationships through chemical modifications and advanced modeling. In addition, evaluating synergistic effects with other inhibitors and conducting long-term safety assessments will be essential to assess therapeutic viability. Importantly, these results suggest that PRSE and its active compounds hold promise for incorporation into functional food formulations aimed at managing metabolic disorders such as type 2 diabetes.

## Data Availability

The original contributions presented in this study are included in the article/Supplementary material, further inquiries can be directed to the corresponding authors.
