# Supplementary material for: α-Glucosidase inhibitory activity of polyphenol-rich sugarcane extract: screening and mechanistic insights based on biolayer interferometry-mass spectrometry
Source: Front Nutr. 2025 Jul 29;12:1575409. doi: 10.3389/fnut.2025.1575409 (PMC12339498; doi:10.3389/fnut.2025.1575409)
Supplement: Supplementary file 1 [file Table_1.docx]

Supplementary Material

# Supplementary Figures


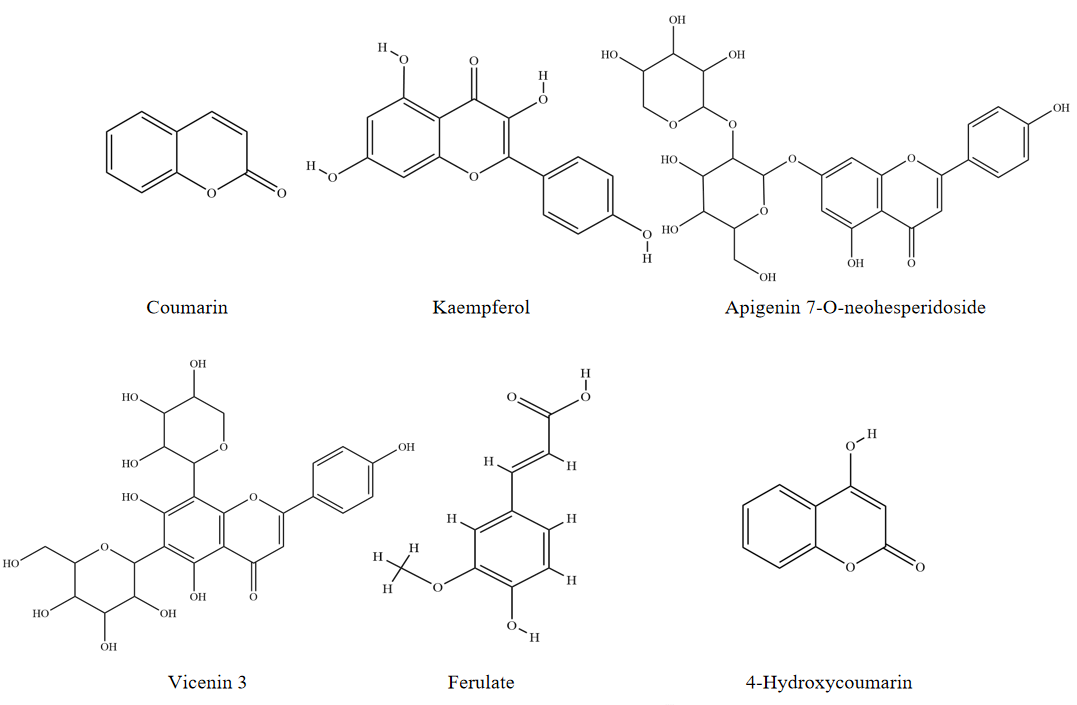


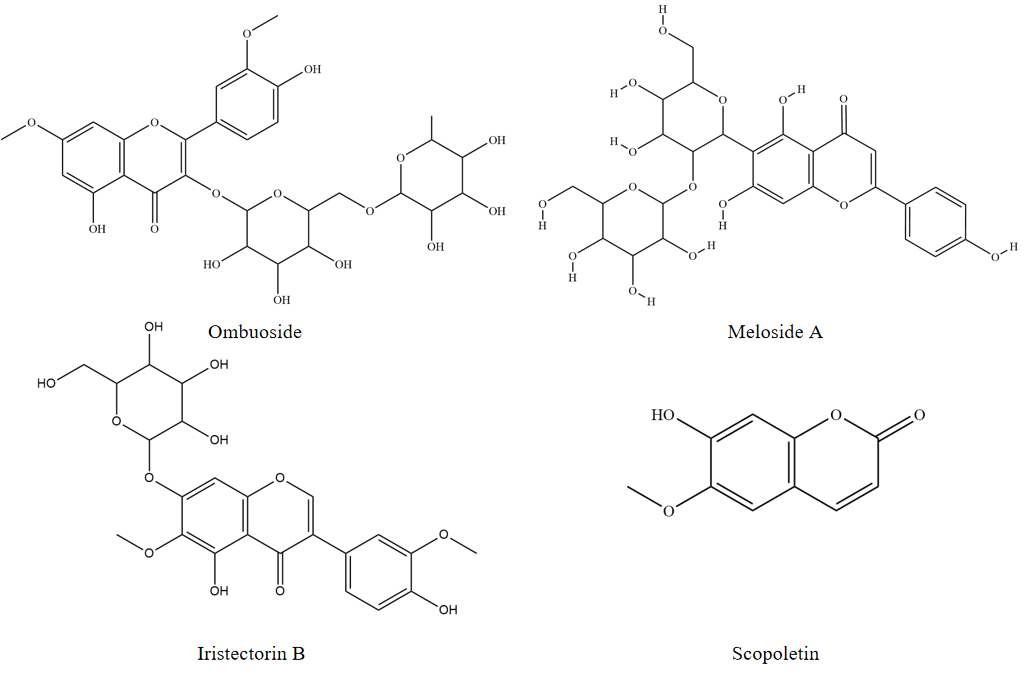


**Supplementary Figure 1.** 2D structure diagrams of the main compounds.


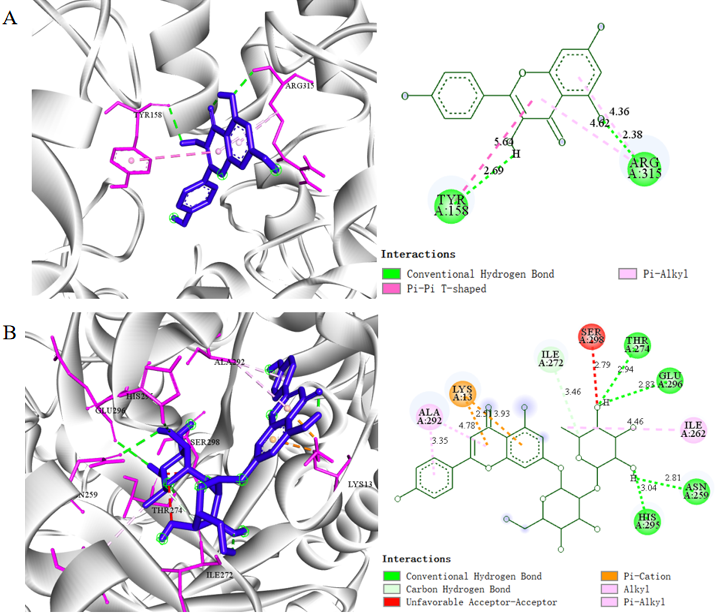


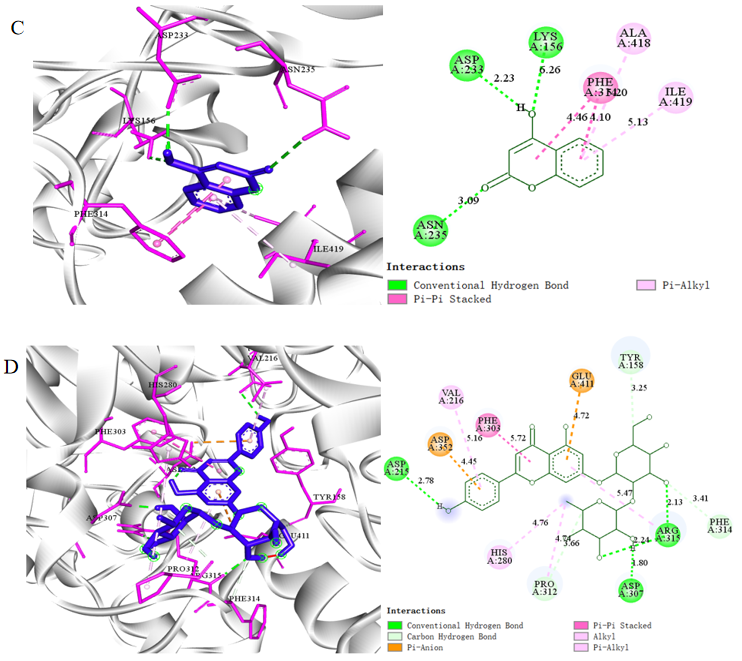


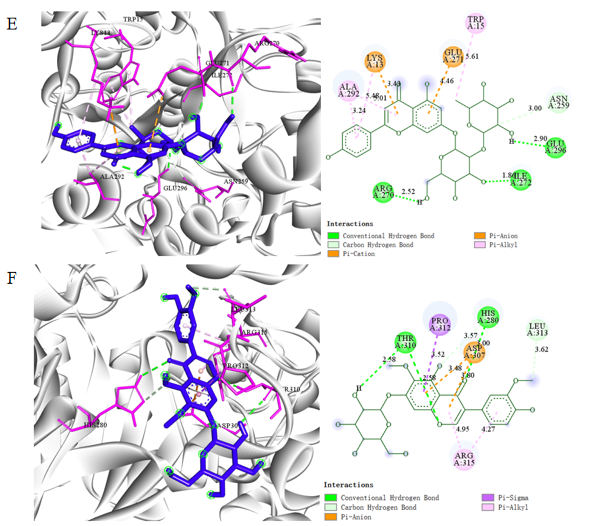


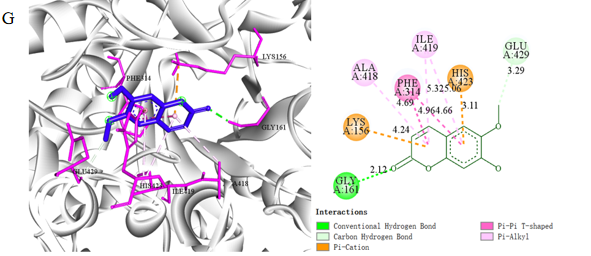


**Supplementary Figure 2.** Molecular docking results: 2D and 3D structures of (A) Kaempferol, (B) Ferulate, (C) 4-Hydroxycoumarin, (D) Ombuoside, (E) Meloside A, (F) Iristectorin B, and (G) Scopoletin with α-glucosidase.

# Supplementary Tables

**Supplementary Table 1.** Molecular docking parameters with acarbose and α-glucosidase.

| ID | Ligands | Affinity (kcal/mol) | Number of HBs | Number of Closest Residues | Interacting Residues |
| --- | --- | --- | --- | --- | --- |
| 1 | Acarbose | -8.1 | 4 | 6 | Arg263, Val266, Lys13, Ile272, Ser298, Asn259 |

# 
